# Sentinel node tumor burden in prediction of prognosis in melanoma patients

**DOI:** 10.1007/s10585-020-10028-0

**Published:** 2020-02-19

**Authors:** Johanna Palve, Leea Ylitalo, Tiina Luukkaala, Juha Jernman, Niina Korhonen

**Affiliations:** 1 Department of Plastic Surgery, Tampere University Hospital and Faculty of Medicine and Health Technology, Tampere University, Teiskontie 35, 33521 Tampere, Finland; 2 Department of Dermatology, Skin Cancer Unit, Helsinki University Central Hospital, Helsinki, Finland; 3 Department of Dermatology and Allergology, Tampere University Hospital and Faculty of Medicine and Health Technology, Tampere University, Tampere, Finland; 4 Research, Development and Innovation Center, Tampere University Hospital and Health Sciences, Faculty of Social Sciences, Tampere University, Tampere, Finland; 5 Department of Pathology, Tampere University and Fimlab Laboratories, Tampere, Finland

**Keywords:** Melanoma, Prognosis in melanoma, Sentinel node-positive, Sentinel node tumor burden

## Abstract

Recent data have demonstrated no survival benefit to immediate completion lymph node dissection (CLND) for positive sentinel node (SN) disease in melanoma. It is important to identify parameters in positive SNs, which predict prognosis in melanoma patients. These might provide prognostic value in staging systems and risk models by guiding high-risk patients’ adjuvant therapy in clinical practice. In this retrospective study of university hospital melanoma database we analyzed tumor burden and prognosis in patients with positive SNs. Patients were stratified by the diameter of tumor deposit, distribution of metastatic focus in SN, ulceration and number of metastatic SNs. These were incorporated in Cox proportional hazard regression models. Predictive ability was assessed using Akaike information criterion and Harrell’s concordance index. A total of 110 patients had positive SN and 104 underwent CLND. Twenty-two (21%) patients had non-SN metastatic disease on CLND. The 5-year melanoma specific survival for CLND-negative patients was 5.00 years (IQR 3.23–5.00, range 0.72–5.00) compared to 3.69 (IQR 2.28–4.72, range 1.01–5.00) years in CLND-positive patients (HR 2.82 (95% CI 1.17–6.76, p = 0.020).The models incorporating distribution of metastatic focus and the largest tumor deposit in SN had highest predictive ability. According to Cox proportional hazard regression models, information criterions and c-index, the diameter of tumor deposit > 4 mm with multifocal location in SN despite of number of metastatic SN were the most important parameters. According to the diameter of tumor deposit and distribution of metastatic focus in SN, adequate stratification of positive SN patients was possible and risk classes for patients were identified.

## Introduction

The standard treatment of melanoma is a wide local excision of primary tumor and sentinel node biopsy (SNB) for staging purposes [1]. The utility of SNB correlates with depth of invasion of the primary tumor and is a routine management in patients with melanoma thicker than 1 mm and may be considered for thin lesions with high-risk characteristics (e.g. ulceration) [2].

Completion lymph node dissection (CLND) has been the standard management approach following positive SNB for melanoma. However, with publications of the German Dermatologic Cooperative Oncology Group (DeCOG) trial [3] and Multicenter Selective Lymphadenectomy-2 trial (MSLT-2) [4], indications for CLND continue to evolve. The latest DeCOG trial results with 72 months follow-up have shown that CLND compared to observation in patients with positive SNB is not associated with increased overall survival [5]. MSLT-2 showed that CLND provides prognostic information as well as improved regional disease control [4], while final analysis of DeCOG-SLT trial concluded that a therapeutic benefit for CLND could not be demonstrated [5]. However, there are differences in SNB protocols and clinicopathologic features of the patient cohorts between centres. Even the conclusions of DeCOG-SLT and MSLT-2 trials have been suggested to be limited by study populations which overall harbored a lower burden of SN disease [6].

Clinical practice has changed dramatically since CLND is expected no longer to be standard procedure after a positive SNB. However, there is a 20% chance of having non-SN (NSN) positivity in CLND after a positive SNB [7]. In cutaneous melanoma patients, the presence of disease in NSNs has been found to be a strong independent adverse prognostic factor for survival [8]. Without CLND the pathologic status of NSNs is not revealed. The systemic therapy for melanoma is evolving rapidly. These therapies have significantly improved the perspective of patients with stage IV melanoma. Since CLND is no longer performed routinely in stage III melanoma, there is a vacuum for these patients for adjuvant therapy. Positive results have been published from clinical trials evaluating the systemic therapy potential also in high-risk stage III melanoma [9]. Thus, it is important to explore variables associated with SN positivity and find patients that are at high-risk of recurrence and/or melanoma-specific death by using information retrieved from the primary melanoma and SNB.

The prior research has identified several micromorphometric criteria to evaluate tumor burden in SN, and a large variety of cut-off metastasis dimensional limits have been studied. Currently, the maximum diameter of the largest tumor deposit (Rotterdam classification) and the intranodal location of SN tumor burden (Dewar classification) are the most common recommended parameters to evaluate tumor burden in SN [1]. Microscopic SN tumor burden (> 1 mm) has already been implemented as an inclusion criterion in some clinical trials. Although this parameter is not yet a formal staging criterion for the N category in the eight edition, documentation of SN tumor burden is an important prognostic factor that will likely guide the development of future prognostic models and validated clinical tools for patients with regional metastatic disease [10]. In various studies, also other parameters have been studied [1, 8, 9, 11–14].

Our study sought to determine an adequate stratification of positive SN patients based on the maximum diameter of the largest tumor deposit, distribution of metastatic focus in SN, ulceration of primary tumor and number of metastatic SN. These parameters might provide accurate prognostic value in staging systems and risk models by guiding high-risk patients’ treatment protocols.

## Materials and methods

According to Tampere university hospital melanoma database, 506 patients underwent successful SNB for cutaneous melanoma between 2006 and 2016. In this retrospective study, we included 110 patients who had positive SNB. Permission to access the clinical records of the melanoma patients for the study was obtained from the scientific center of Tampere University Hospital. In our skin cancer unit, we have 10-year follow-up program (three clinical check-up visits/year) for the melanoma patients with metastasis in SN. In this study, we included patients with new melanoma diagnosed and operated between 1.1.2006 and 31.12.2016. The follow-up was performed until 31.1.2019. Patients were censored from survival analyses at 5-year time point. In future, we will continue the follow-up with these patients to have 10-year follow-up for all.

Retrospective review of records was performed to determine following information: age, gender, primary tumor site (head/neck, trunk, upper limb, lower limb), tumor characteristics (Breslow thickness, ulceration, subtype superficial spreading melanoma, (SSM), nodular melanoma (NM), lentigo maligna melanoma (LMM) and acral melanoma (AM)) and time of diagnosis. Clinical outcomes regarding sentinel node (SN) included the lymph node basin (cervical, axillary, inguinal and bi-locational), maximum size (in millimeters) of largest tumor deposit in SN, distribution of metastatic foci in SN subcapsular, parenchymal and multifocal, number of SNs removed and number of metastatic SN. Clinical outcomes regarding CLND included number of nodes harvested in CLND and number of metastatic lymph nodes found on CLND. Time of the recurrence was recorded as well as time of death and reason for death (melanoma, other).

Stratifying patients according to the largest tumor deposit (< 1 mm, 1–4 mm or > 4 mm), distribution of metastatic focus in SN [unifocal (subcapsular or parenchymal) or multifocal] and ulceration (absent or present) resulted in three categories of parameter pairs: ulceration and distribution of metastatic focus, ulceration and largest tumor deposit, distribution of metastatic focus and largest tumor deposit. Each three categories were further divided to groups: group 1 (ulceration absent and tumor deposit < 1 mm/1–4 mm/ > 4 mm, ulceration present and tumor deposit < 1 mm/1–4 mm/ > 4 mm), group 2 (ulceration absent and tumor distribution uni-or multifocal, ulceration present and tumor distribution uni-or multifocal), group 3 (tumor distribution unifocal and tumor deposit < 1 mm/1–4 mm/> 4 mm, tumor distribution multifocal and tumor deposit < 1 mm/1–4 mm/> 4 mm).

We further analyzed parameter pairs of the largest tumor deposit (< 1 mm, 1–4 mm or > 4 mm)/subcapsular and parenchymal location of metastatic focus (group 4) and the largest tumor deposit (< 1 mm, 1–4 mm or > 4 mm)/number of metastatic SN (1 or > 1) (group 5).

SNB was performed for all patients with a melanoma with Breslow thickness > 1 mm or 0.75–1 mm with ulceration and/or mitotic activity > 1/mm^2^. A preoperative lymphoscintigraphy was performed for all patients using 99mTc labeled human albumin colloid injected intra-dermally. Patent blue was used until October 2013. Single-photon emission computerized tomography/computed tomography (SPECT/CT) was performed for all melanoma patients preoperatively starting in October 2013. Intraoperative identification of the SN was done with a handheld gamma probe. Radioactive nodes that had count > 10% of the most radioactive node were also considered as SNs. Histopathologic analysis of SN consisted of sectioning and staining with hematoxylin and eosin. The immunohistochemical markers S-100, Melan-A and HMB-4 were used routinely.

### Statistical analysis

Predictive factors for SN were calculated by univariable using Mann–Whitney test, Pearson chi-square test or Fisher’s exact test. Disease-free survival (DFS) was defined as the time between the time of diagnosis and detected recurrence. Survival was calculated from the time of diagnosis to death, either from melanoma (melanoma-specific survival, MSS) or other causes. Time-to-event analyses were performed using Cox proportional hazard regression analysis. A p-value of < 0.05 was considered significant. Harrell’s concordance index (c-index) and the Akaike information criterion (AIC) values were analysed to compare the predictive ability of the different Cox regression models [15, 16]. A higher c-index and lower AIC value indicate a better model for predicting outcome. All analyses were performed using IBM SPSS Statistics for Windows, version 25.0 software (IBM Corp., Armonk, NY) and R (version 3.6.1, R Foundation for Statistical Computing, Vienna, Austria, 2019).

## Results

From 2006 to 2016, 506 patients [274 (54%) men and 232 (46%) women] underwent SNB. A total of 110 patients out of 506 (22%) had metastasis in SNB and were included in the study. Of these 110 SNB positive cases, six (5%) had isolated tumor cells (ITC) and CLND was not performed. Follow-up was performed until 31.1.2019. Median follow-up time (n = 104) 4.71 (IQR 2.92–7.85, range 0.72–12.02), CLND negative n = 82 Md 5.29 (IQR 3.23–8.22, range 0.72–12.02) and CLND positive n = 22 Md 3.69 (IQR 2.28–4.90, range 1.01–9.21). There was a difference in the length of follow-up period between subgroups (Mann–Whitney test p = 0.016).

### CLND-positive compared with CLND-negative cases

We compared the characteristics of the CLND-positive patients and tumors to those of the CLND-negative group (Table 1). CLND-positive patients were slightly younger (p = 0.349), more male predominant p = 0.107), had more commonly melanomas thicker than 2 mm (p = 0.052) and ulcerated melanomas (p = 0.222) than CLND-negative patients. SSM was the most common subtype in both groups, but NM was more common and AM less common in CLND-negative patients than in CLND-positive patients (p = 0.064). Trunk and lower limb were the most common locations of melanoma in both groups, but head/neck area was more common and upper limb less common location in CLND-positive patients (p = 0.102). SN basin was more commonly in axillary region and less commonly in cervical area in CLND-negative patients compared to CLND-positive patients (p = 0.102). Number of SNs removed did not differ significantly between groups. Of CLND negative patients, 29 received interferon treatment compared to nine CLND positive patients (p = 0.632). Six patients out of 15 with bi-locational SN basin had metastases in two basins.

**Table 1:** Patient and tumor characteristics overall and by CLND status

	CLND negative n = 82	CLND positive (n = 22)	p-value
	n (%)	n (%)	
Age (years), mean (SD)	66.5 (12.9)	63.4 (16.5)	0.349
Sex, n (%)			0.107
Men	48 (59)	17 (77)	
Women	34 (41)	5 (23)	
Ulceration, n (%)			0.222
No	31 (38)	5 (23)	
Yes	51 (62)	17 (77)	
Tumor thickness (mm), n (%)			0.052
Br < 2 mm	24 (29)	2 (9)	
Br ≥ 2 mm	58 (71)	20 (91)	
Subtype, n (%)			0.064
SSM	45 (55)	13 (59)	
NM	33 (40)	5 (23)	
AM	4 (5)	4 (18)	
Tumor location, n (%)			0.102
Head and neck	7 (8)	6 (27)	
Trunk	36 (44)	8 (36)	
Upper limb	18 (22)	2 (9)	
Lower limb	21 (26)	6 (27)	
Number of SN removed, n (%)			0.899
1	14 (17)	4 (18)	
2	30 (37)	9 (41)	
≥ 3	38 (46)	9 (41)	
Sentinel node location, n (%)			0.125
Axillary	39 (48)	6 (27)	
Inguinal	24 (29)	6 (27)	
Cervical	8 (10)	6 (27)	
Bi-locational	11 (13)	4 (18)	
Interferon	29 (35)	9 (41)	0.632
Size of largest metastatic focus, n (%)			0.349
< 1 mm	27 (33)	5 (23)	
1–4 mm	34 (41)	8 (36)	
> 4 mm	21 (26)	9 (41)	
Distribution of metastatic focus/foci			0.240
Subcapsular	32 (39)	6 (27)	
Parenchymal	31 (38)	7 (32)	
Multifocal	19 (23)	9 (41)	
Number of metastatic sentinel nodes			0.206
One	65 (79)	14 (64)	
Two	14 (17)	6 (27)	
Three or more	3 (4)	2 (9)	
Type of recurrence			0.600
No recurrence	43 (52)	9 (41)	
Local recurrence	7 (8)	3 (14)	
Regional recurrence	7 (8)	3 (14)	
Distant metastasis	25 (31)	7 (32)	
Exitus during follow-up, n (%)			0.411
Alive	49 (60)	10 (46)	
Of melanoma	22 (27)	9 (41)	
Of other reasons	11 (13)	3 (14)	
Follow-up time (years), median (range)	5.29 (0.72–12.02)	3.69 (1.01–9.21)	0.016

Univariable analyses were performed using Mann–Whitney test, Pearson chi-square or Fisher’s exact test. In multivariable logistic regression analysis, adjusting factors (age, sex, categorized tumor thickness and tumor location) were included simultaneously into the model. Results were shown by odds ratios (OR) with 95% confidence intervals (CI)

*SSM* superficial spreading melanoma, *NM* nodular melanoma, *AM* acral melanoma, *CLND positive* residual disease in lymph nodes in complete lymph node dissection, *CLND negative* no residual disease in lymph nodes in complete lymph node dissection

CLND-negative patients had more commonly one metastatic SN and less commonly two or more metastatic SNs than CLND-positives (p = 0.206). SN tumor burden located multifocally (p = 0.240) and maximum diameter of the largest tumor deposit > 4 mm (p = 0.349) was more likely in CLND-positive patients. Detailed data of CLND-positive cases is presented in Table 2.

**Table 2:** Detailed data from CLND positive cases (n = 22)

Age (years)	Sex	Ulc	Br (mm)	Type	Tumor loc	SN loc	N of SN	Size of focus (mm)	Distribution of focus	N of met SN	Evac met	Type of recurrence	Exitus
56	W	Yes	2.3	NM	Tr	Ax	2	4.2	Multifocal	1	2	Distant	Melanoma
72	W	Yes	3.0	AM	LL	Ing	2	2.3	Multifocal	1	3	Distant	Melanoma
72	W	No	2.1	SSM	HN	Cer	4	5.7	Multifocal	3	3	Distant	Melanoma
67	M	Yes	3.5	SSM	Tr	Ax	5	4.3	Multifocal	1	1	Distant	Melanoma
83	M	Yes	9.0	AM	LL	Ing	1	7.2	Multifocal	1	4	Distant	Melanoma
74	M	Yes	6.5	NM	HN	Cer	5	2.5	Multifocal	1	3	Distant	Melanoma
68	M	No	2.9	SSM	HN	Cer	3	3.0	Multifocal	3	4	Local	Melanoma
53	M	Yes	1.0	SSM	Tr	Ax	1	6.3	Multifocal	1	4	Regional	No
61	M	Yes	4.0	AM	LL	Ing	4	5.8	Multifocal	2	2	No	No
88	W	Yes	2.5	SSM	Tr	Bi	2	5.1	Parenchymal	1	7	Regional	Other
21	M	Yes	3.9	NM	HN	Cer	2	1.0	Parenchymal	2	2	No	Other
88	M	Yes	5.0	SSM	HN	Cer	2	8.0	Parenchymal	1	1	No	Other
55	M	Yes	2.5	AM	LL	Ing	1	0.7	Parenchymal	1	1	Distant	Melanoma
75	M	Yes	9.0	NM	HN	Cer	3	1.1	Parenchymal	1	1	No	No
49	M	No	2.7	SSM	LL	Ing	3	4.9	Parenchymal	2	2	Local	No
36	M	Yes	4.0	SSM	Tr	Bi	4	1.0	Parenchymal	2	2	No	No
67	W	Yes	5.0	SSM	LL	Bi	2	0.5	Subcapsular	2	5	Local	No
71	M	Yes	5.0	SSM	Tr	Ing	2	3.0	Subcapsular	1	1	No	No
77	M	Yes	2.2	SSM	UL	Ax	2	2.0	Subcapsular	1	1	No	No
52	M	No	1.0	SSM	UL	Ax	3	0.6	Subcapsular	1	1	No	No
63	M	No	8.0	SSM	Tr	Ax	1	0.4	Subcapsular	1	1	No	No
81	M	Yes	12.0	NM	Tr	Bi	2	0.7	Subcapsular	2	1	Regional	Melanoma

Median follow-up time for CLND positive patients was 3.69 years (IQR 2.28–4.90), range 1.01–9.21

*CLND positive* residual disease in lymph nodes in complete lymph node dissection, *w* woman, *m* man, *Br* Breslow thickness, *SSM* superficial spreading melanoma, *NM* nodular melanoma, *AM* acral melanoma., *tumor loc* primary tumor location, *Tr* trunk, *LL* lower limb, *HN* head and neck, *UL* upper limb, *SN loc* SN basin, *Ax* axillary, *Ing* inguinal, *Cer* cervical, *Bi* bilocational, *size of focus* the maximum diameter of tumor deposit, *distribution of focus* location of tumor deposit in SN, *N of met SN* number of metastatic SN, *evac met* number of metastatic lymph nodes in CLND

### Recurrence patterns and survival analysis

A half (43/82, 52%) of CLND-negative compared to 41% (9/22) of CLND-positive patients did not have recurrence during the study period (Table 1). Both groups had equal proportion of local and regional recurrences and distant metastases. In CLND-positive patients recurrences occurred earlier with 5-year disease free survival (DFS) median of 2.18 years (IQR 0.77–3.75, range 0.30–5.00) compared to 3.83 years (IQR 1.87–5.00, range 0.15–5.00) in CLND-negative patients (HR 1.79 (95% CI 0.92–3.45, p = 0.084).

The 5-year melanoma specific survival (MSS) for CLND-negative patients was 5.00 years (IQR 3.23–5.00, range 0.72–5.00) compared to 3.69 (IQR 2.28–4.72, range 1.01–5.00) years in CLND-positive patients (HR 2.82 (95% CI 1.17–6.76, p = 0.020). The 5-year cumulative proportion surviving at time for CLND negative was 80.4% (Std. error 4.8%) and for CLND positive 41.6% (Std. error 13.8%).

### Stratification models and risk classes

**Fig. 1:**
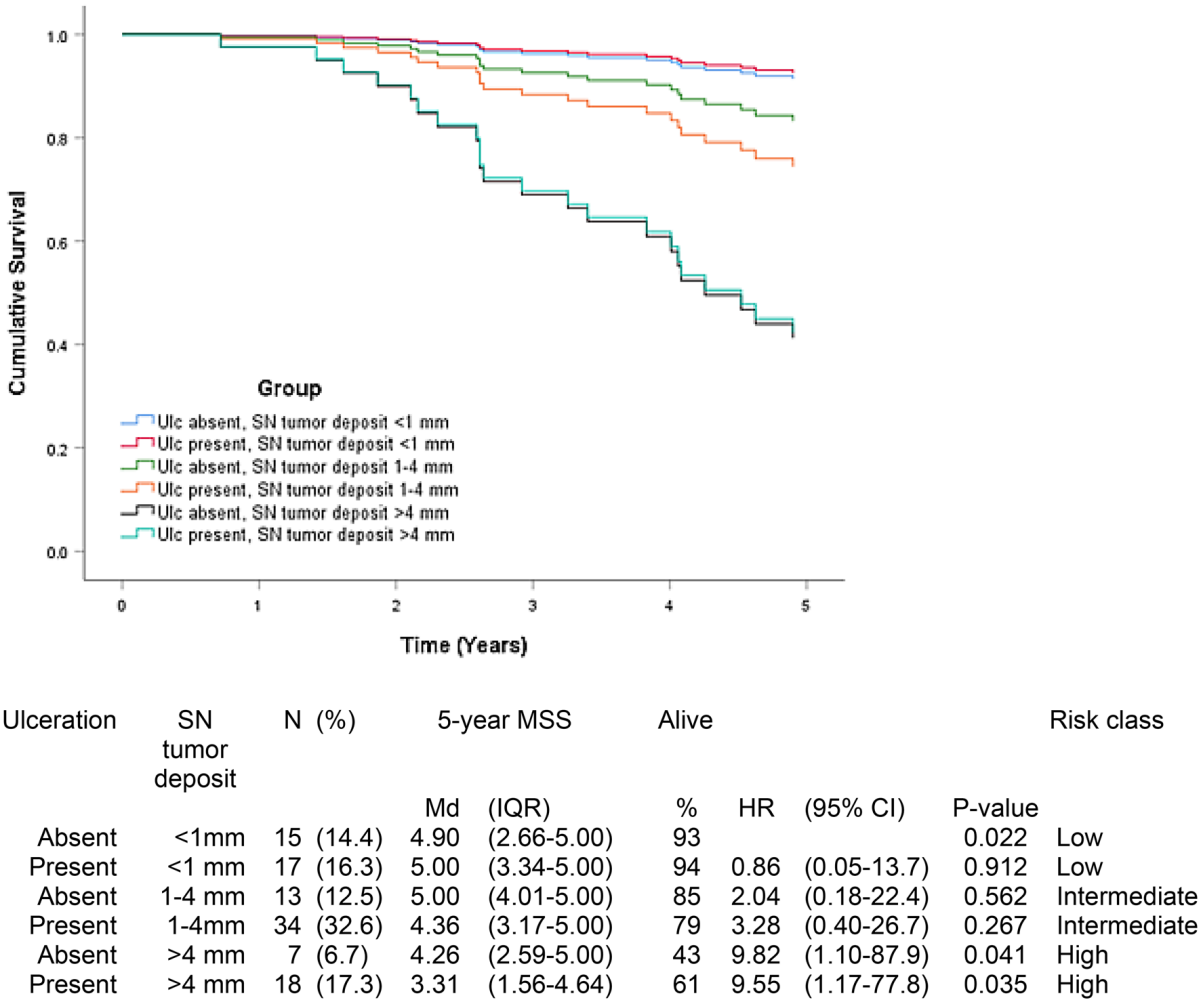
Five-year melanoma specific survival in group 1 (N = 104). In this group, the largest tumor deposit and ulceration status of the tumor were incorporated in the model. The Bayesian information criteria of the model was 190.7 and concordance index = 0.387. A total of 22 (21.2%) of the patients were deceased

**Fig. 2:**
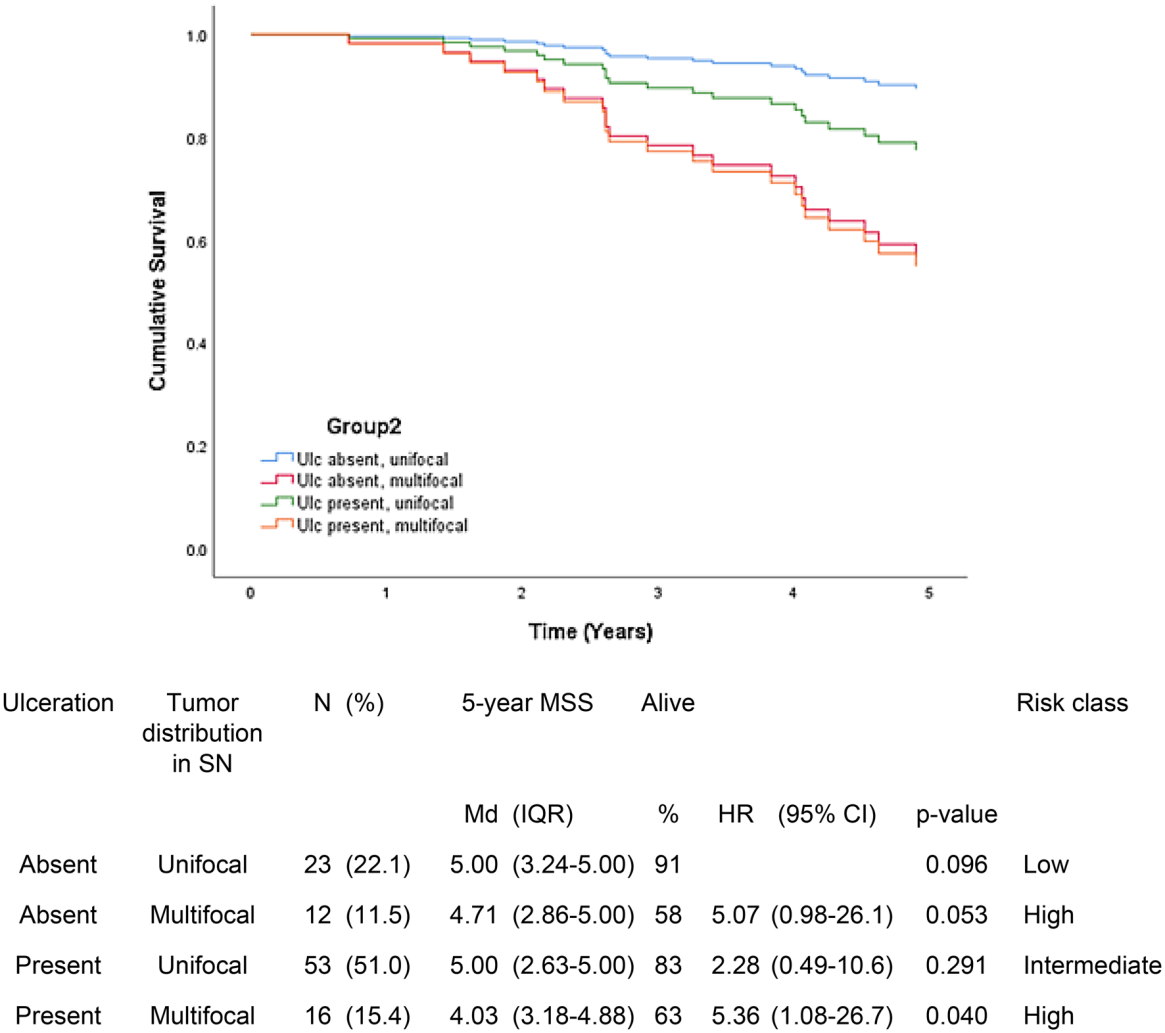
Five-year melanoma specific survival in group 2 (N = 104). In this group, the ulceration status of the tumor and distribution of metastatic focus in sentinel node were incorporated in the model. The Bayesian information criteria of the model was 192.3 and concordance index = 0.670. A total of 22 (21.2%) of the patients were deceased

**Fig. 3:**
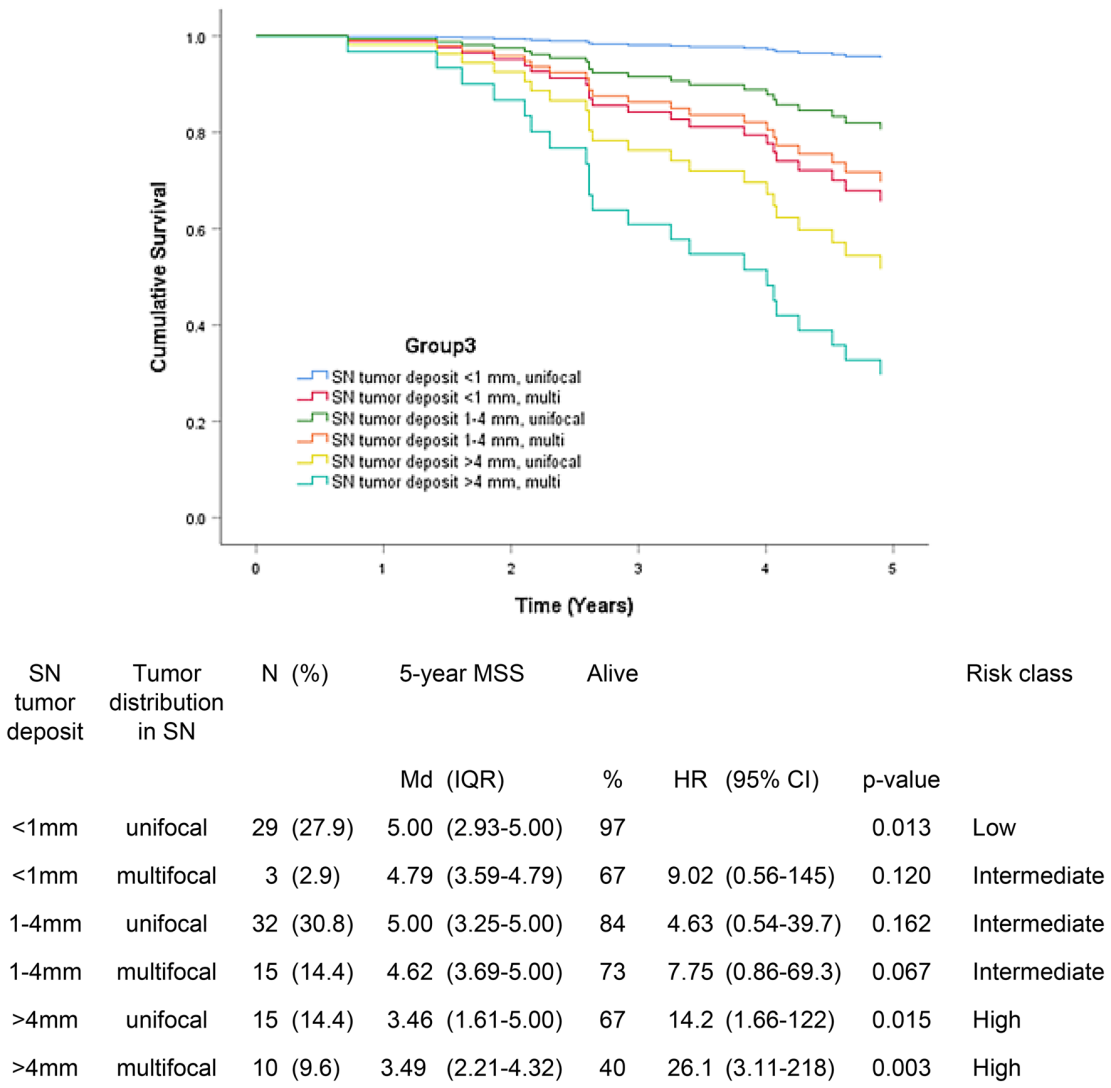
Five-year melanoma specific survival in group 3 (N = 104). In this group, the largest tumor deposit and the distribution of metastatic focus in sentinel node were incorporated in the model. The Bayesian information criteria of the model was 187.5 and concordance index = 0.408. A total of 22 (21.2%) of the patients were deceased

**Fig. 4:**
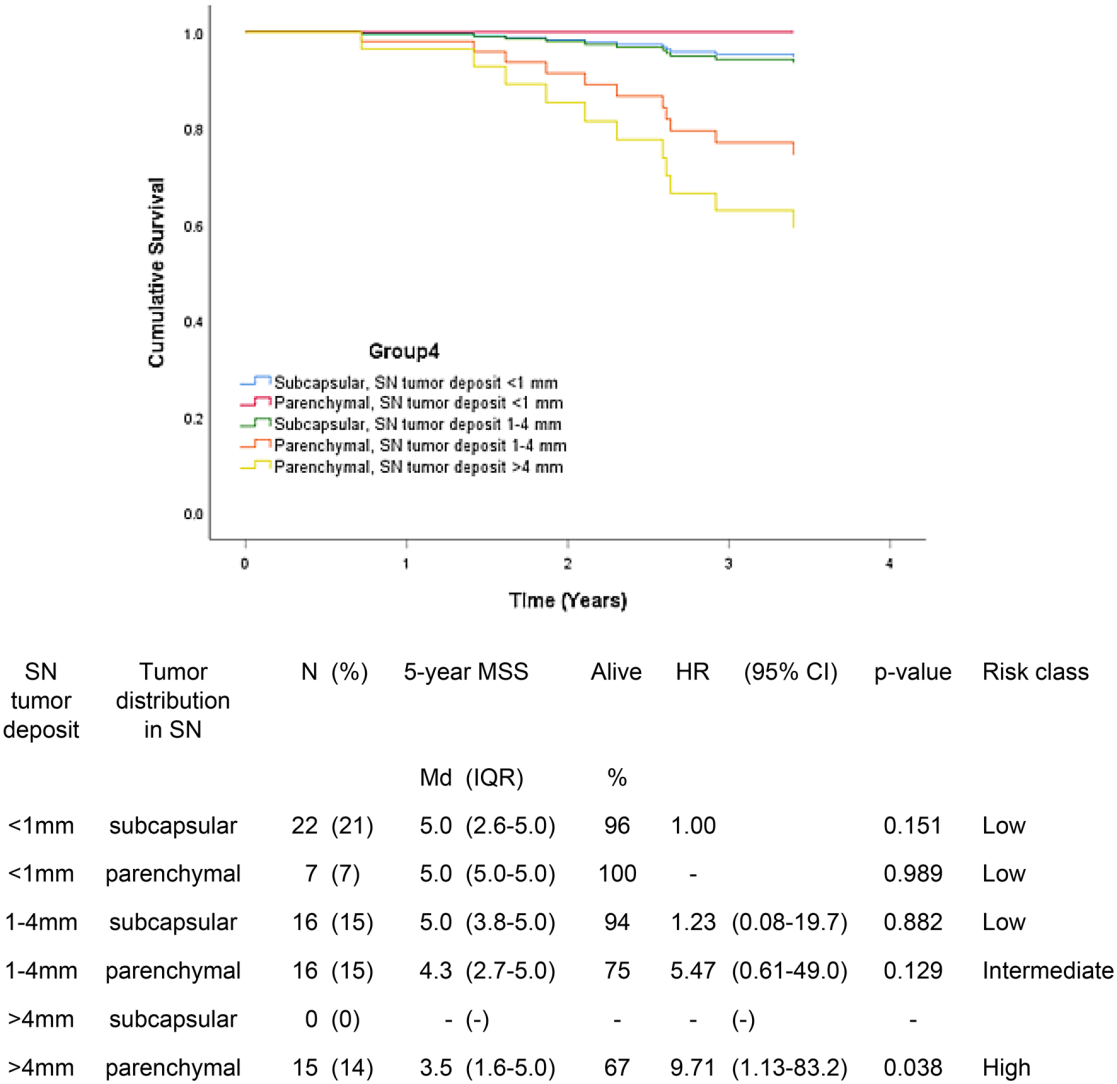
Five-year melanoma specific survival in group 4 (N = 76). In this group, the location of metastatic focus and the largest tumor deposit were incorporated in the model. The Bayesian information criteria of the model was 90.4 and concordance index = 0.424. A total of 11 (10.6%) of the patients were deceased

**Fig. 5:**
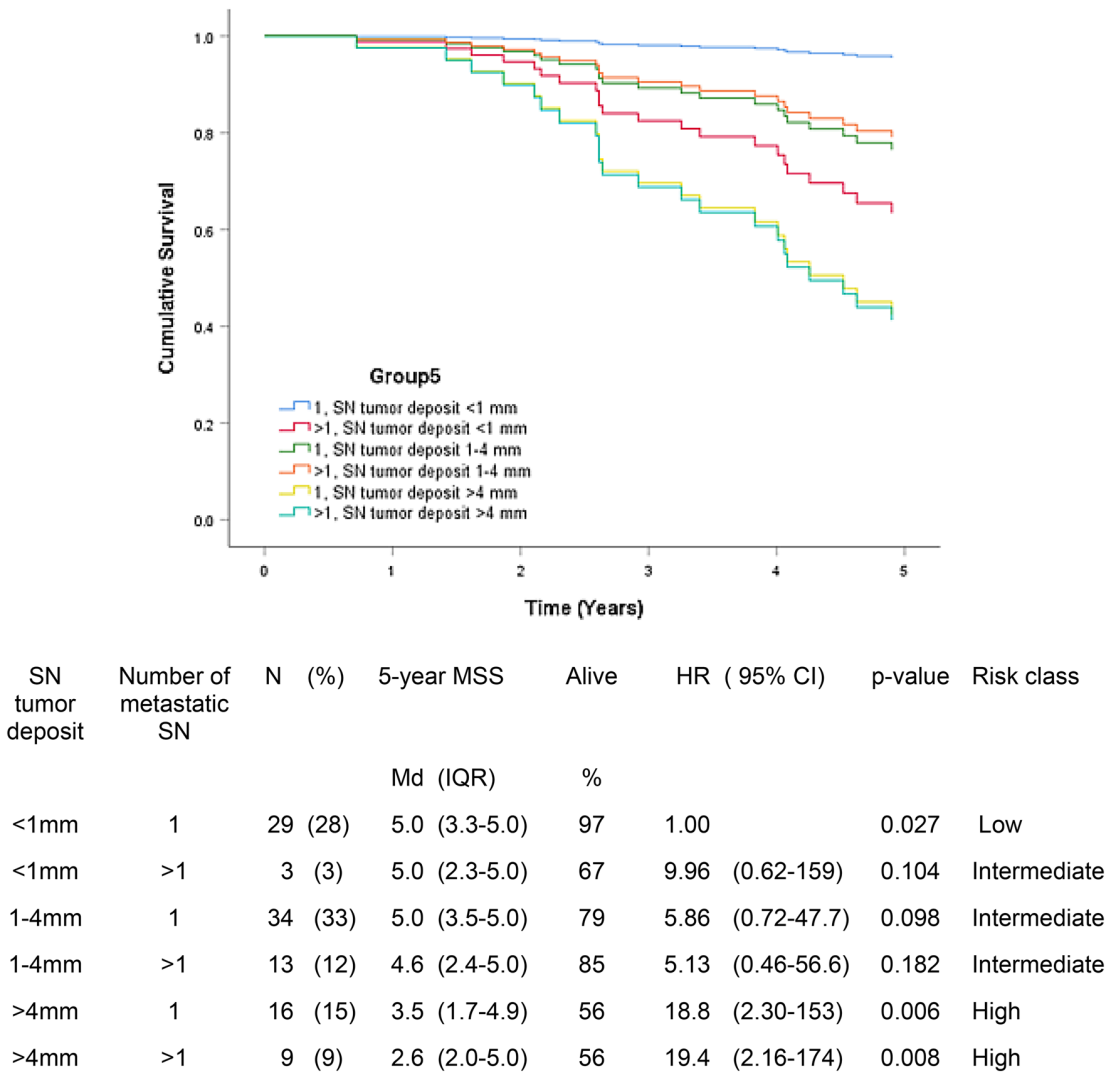
Five-year melanoma specific survival in group 5 (N = 104). In this group, the number of metastatic sentinel node and the largest tumor deposit were incorporated in the model. The Bayesian information criteria of the model was 188.9 and concordance index = 0.378. A total of 22 (21.2%) of the patients were deceased

The 5-year MSS was different inside groups. The distribution, model performance and 5-year MSS per group and the corresponding Cox proportional hazard regression models are presented in Figs. 1, 2, 3, 4 and 5.

In group 1, patients with tumor deposit < 1 mm and ulceration in primary tumor had 5-year MSS median of 5.00 years (94%) compared to 3.31 years (61%) in patients with tumor deposit > 4 mm and ulcerated primary tumor (p = 0.035) (Fig. 1). In patients with tumor deposit > 4 mm ulceration status of primary tumor did not have influence in survival.

In group 2, patients with unifocal distribution of tumor deposit and primary melanoma without ulceration had 5-year MSS median of 5.00 years (91%) compared to 4.03 years (63%) in patients with ulcerated primary tumor and multifocal distribution of tumor deposit in SN (p = 0.040) (Fig. 2). Multifocality clearly influenced the prognosis despite of ulceration status of primary tumor.

In group 3, patients with unifocal tumor distribution and tumor deposit < 1 mm had 5-year MSS median of 5.00 years (97%) compared to 3.49 years (40%) in patients with multifocal tumor distribution and tumor deposit > 4 mm in SN (p = 0.002) (Fig. 3). Unifocal tumor location seemed to be associated with better prognosis.

In group 4, patients with tumor deposit < 1 mm either with subcapsular or parenchymal tumor location and patients with tumor deposit 1–4 mm with subcapsular location had 5-year MSS median of 5.0 years (94–100%) compared to 3.5–4.3 years (67–75%) in patients with parenchymal tumor location either tumor deposit with 1–4 mm or > 4 mm. Parenchymal location did not worsen the prognosis in patients with tumor deposit < 1 mm, but with increasing tumor deposit it predicted worse survival. Subcapsular location seemed to be associated with better prognosis in patients with tumor deposit with 1–4 mm.

In group 5, patients with tumor deposit < 1 mm and with one metastatic SN had 5-year MSS 5.0 (97%) compared to 2.6 years (56%) in patients with tumor deposit > 4 mm and more than one metastatic SN (p = 0.008). In patients with tumor deposit > 4 mm, the number of metastatic SN did not have great influence in survival.

According to these differences in survival, we were able to find possible risk classes inside groups (low, intermediate and high).

## Discussion

In this study, we evaluated five models with four parameters, including the maximum diameter of the largest tumor deposit, distribution of metastatic focus in SN, ulceration of primary tumor and number of metastatic SN, in melanoma patients with positive SN to investigate the possible relationship with patient survival. We were able to determine an adequate stratification and identify risk classes for patients.

SNB is the most accurate staging tool for melanoma patients [17]. The 5-year overall survival is about 90% for patients with negative SN and 71% for patients with positive SN [1]. Approximately 20% of melanoma patients harbor metastases in NSNs after a positive SNB [18]. In cutaneous melanoma patients, the presence of disease in NSNs has been found to be a strong independent adverse prognostic factor for survival [8]. Also in our study, the worse 5-year MSS was also seen in CLND-positive patients compared to CLND-negative patients. Positive SNs in two basins has been published to be worse prognostic factor [19, 20]. In our study, only six patients had positive SN in two basins.

There have been efforts to identify predictors for CLND positivity and prognosis focusing on characteristics of the SN metastasis and histologic and anatomic characterization of the primary tumor, which may carry additional prognostic value [11]. In terms of anatomic location, there has been variable reports showing that lower extremity primary tumors are more likely than both truncal and head and neck tumors in patients with NSN-positivity [21], while other showed that primary tumor in limbs conferred to a lower risk of NSN-positivity compared to trunk and head and neck [18]. In our study, trunk and lower limb were the most common locations of melanoma in both groups, but head and neck area was more common and upper limb less common location in CLND-positive patients. SN basin has also been investigated as prognostic parameter of NSN-positivity. It has been suggested that axillary bed is less likely than either groin or cervical basins to yield positive NSNs [14], which was also shown in our study.

The most used tumor burden parameters are the maximum diameter of the SN metastasis and microanatomic location of metastasis in the SN [17]. In addition to diameter and location, we also incorporated the number of metastatic SNs and ulceration of the primary tumor into the models. These parameters were included because in overall characteristics of our study cohort by CLND status, CLND-positive patients had more commonly ulcerated tumors and more than one metastatic SN than CLND-negative patients. Previous studies have demonstrated that 70%- 80% of patients with positive SN will have only a single positive node [12], which is in agreement with our study. Patients with a single positive node are clinically stage III according to AJCC 8th edition and thus candidates for adjuvant immunotherapy, but they are suggested to represent the lowest risk group for NSN metastases and might be ones in whom adjuvant therapy can be safely avoided [10]. In our model incorporating the number of metastatic SN and maximum diameter of tumor deposit, the patients with tumor deposit < 1 mm and one metastatic SN had the best 5-year MSS. The diameter of tumor deposit influenced more in prognosis than the number of metastatic SN. Number of positive SNs did not have apparent impact either in prediction of positive NSNs in study by Gershenwald et al. [21].

Ulceration is an adverse prognostic factor in cutaneous melanoma [10]. It has been shown to be the most important predictor of lymphatic involvement especially in thick melanomas [13]. Ulceration has also been evaluated in models associated with prognostic role in melanoma [9, 13]. In our study, ulceration was incorporated with tumor deposit diameter and distribution in SN. Ulceration predicted worse survival in patients with tumor deposit of 1–4 mm, while in patients with tumor deposit < 1 mm and > 4 mm ulceration did not play significant role. In the model incorporating ulceration with tumor distribution, the multifocal location of tumor deposit influenced more in survival than ulceration. In prior studies, primary tumor ulceration had no apparent impact either on prediction of positive NSNs [21].

We investigated the combination of the maximum diameter of the SN metastasis and microanatomic location of metastasis in the SN in two models. In first model, we incorporated maximum diameter of the largest tumor deposit with unifocal or multifocal location of tumor deposit tumor in SN. In the second model, we further evaluated the model incorporating the diameter of tumor deposit and subcapsular or parenchymal location of tumor deposit in SN. In prior studies, cut points and measures of SN disease burden vary with each reported model. We chose 1 mm diameter to the cut point because in earlier studies, maximum tumor size > 1 mm has been suggested to be the most reliable parameter associated with higher NSN-positivity and poorer MSS [1, 9, 11, 22]. Multifocal SN tumor deposit location has been shown to be independently associated with NSN-positivity [11, 12] and subcapsular site with positive prognostic significance [1]. In our models, tumor diameter < 1 mm was clearly associated with better and diameter > 4 mm to poorer survival. In patients with tumor deposit diameter of 1–4 mm, the tumor distribution seemed to influence more in survival. Multifocality and parenchymal location of tumor deposit worsen the survival.

In our study, the risk of non SN involvement was higher when (1) the primary melanoma is thicker than 2 mm, (2) ulcerated, (3) more SNs involved; and (4) the largest metastatic focus in SN is > 1 mm or (5) is located multifocally or in parenchyme. The results of the current study are largely consistent with previously published literature. Ulceration of primary tumor and number of metastatic SN had less impact in stratification, while the combination of the maximum diameter of the largest tumor deposit and distribution of metastatic focus in SN yielded the best stratification. These parameters might provide accurate prognostic value in staging systems and risk models by guiding high-risk patients’ treatment protocols and finding ones in whom adjuvant therapy can be safely avoided. However, stratifying for survival is not necessarily equivalent to stratifying for therapeutic benefit. Whether the presented risk classes are able to stratify accurately for therapeutic benefit as well, needs to be evaluated. On the other hand, risk stratification can also help to inform the frequency of ultrasound nodal surveillance in the SN basin, with lower risk patients perhaps requiring less frequent ultrasound imaging and higher risk patients requiring a more intense surveillance program.

This study has certain limitations. One of the limitations is its retrospective design. The patient cohort upon which the model was based consisted of a relatively small cohort. The analyses were, however, based on complete case analysis without missing data.

## Conclusion

In this study, we evaluated our experience of SN-positive patients undergoing CLND to identify those at high or low risk. By using the easy-to-obtain clinicopathological information, including diameter of tumor deposit and distribution of metastatic focus in SN we were able to identify risk classes for patients. SN tumor burden characterization provides prognostic information, which is useful in patients who do not undergo CLND. These parameters might provide accurate prognostic value in staging systems and risk models by guiding high-risk patients’ treatment protocols and finding ones in whom adjuvant therapy can be safely avoided.

## Abbreviations

CLND: Completion lymph node dissection
SN: Sentinel node
SNB: Sentinel node biopsy
SSM: Superficial spreading melanoma
NM: Nodular melanoma
LMM: Lentigo maligna melanoma
AM: Acral melanoma
MSS: Melanoma specific survival
NSN: Non-sentinel node
